# Deep eutectic solvent in water pickering emulsions stabilised by cellulose nanofibrils†‡

**DOI:** 10.1039/d0ra07575b

**Published:** 2020-10-07

**Authors:** Saffron J. Bryant, Marcelo A. da Silva, Kazi M. Zakir Hossain, Vincenzo Calabrese, Janet L. Scott, Karen J. Edler

**Affiliations:** Department of Chemistry, University of Bath Claverton Down Bath BA2 7AY UK K.Edler@bath.ac.uk; School of Science, RMIT University Melbourne Victoria 3001 Australia saffron.bryant@rmit.edu.au; Centre for Sustainable Chemical Technologies, University of Bath Claverton Down Bath BA2 7AY UK

## Abstract

Deep eutectic solvent (menthol : dodecanoic acid) in water (30 : 70) emulsions stabilised with partially oxidised cellulose nanoparticles remained stable for 200 days at room temperature. Deep eutectic-based emulsions offer potential for non-aqueous reaction systems, chemical extraction, and controlled release. Pickering emulsions using polysaccharides are less toxic and more stable than surfactant-stabilised emulsions.

## Abstract 2

Deep eutectic solvent in water emulsions stabilised with bio-macromolecules were stable for more than 200 days.

## Graphical abstract

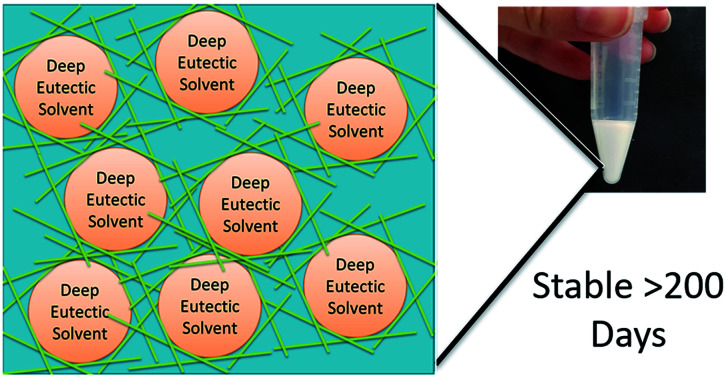

## Introduction

Deep eutectic solvents (DESs) are a promising new type of non-volatile solvent composed of hydrogen bond donors and acceptors. They are a highly tuneable subclass of ionic liquid (IL) but are often cheaper, easier to make, and more environmentally friendly.^1–4^

DESs and the broader ionic liquid (IL) solvent class have potential as reaction media and reactor systems.^2,5–9^ Their tuneable properties, including temperature stability and solvation of drug and catalytic molecules make ILs and DESs preferable to traditional oil/water emulsions.^2,10,11^ There have been some reports of ionic liquid (IL) based emulsions (using IL as an additive, or as the oil, or as the polar phase, or even IL in IL emulsions).^10–13^ There are also reports of DES in oil emulsions, stabilised with conventional surfactants.^14^ However, to the authors' knowledge, the only report of a DES in water emulsion comes from the commercial EMLA® cream (EMLA stands for Eutectic Mixture of Local Anesthetics) which uses a eutectic mixture of lidocaine and prilocaine, stabilised by a non-ionic surfactant.^15^

The DES in water pickering emulsion reported here has an advantage over these previously reported formulations because it does not use expensive and potentially toxic surfactants. In addition, as water is the major phase, it is more compatible with applications that require low toxicity and low cost, than DES in oil emulsions.

Menthol : dodecanoic acid (2 : 1) is a hydrophobic DES with a melting point around 7 °C.^16^ It has potential for extraction of materials from water,^17,18^ separation of metals,^19,20^ and extraction of pesticides from water.^21^

TEMPO-oxidised cellulose nanofibrils (OCNF) are negatively charged nanoparticles that can form Pickering-type emulsions.^22–24^ Pickering emulsions stabilised by other polysaccharide-based nanoparticles have also been reported.^25–27^ However, until now, their use has been limited to emulsions using model oils (such as hexadecane), or sunflower oil.

Emulsions containing 30 vol% menthol : dodecanoic acid were stabilised using either OCNF, or hydrophobically modified OCNF (C8-OCNF). These emulsions, including their stability over time were assessed using laser diffraction, visual observation, and small-angle X-ray scattering.

## Experimental

### Oxidised cellulose nanofibrils

Oxidised cellulose nanofibrils provided by Croda® as a *ca.* 8 wt% solids paste in water were prepared *via* TEMPO-mediated oxidation as previously described.^28,29^ Previous work by this group determined the degree of oxidation for this particular batch of fibrils to be 25% *i.e.* number of carboxylate groups compared to total anhydroglucose units.^30,31^ The fibrils have a large aspect ratio (hundreds of nm in length and a cross section of ∼5 nm)^32^ and a high negative surface charge (−60 mV in *ζ* potential).^28–30^

Residual salts and preservatives were removed from OCNF by dialysis against deionised water (18.2 MΩ cm) as previously described.^32^ The OCNF was then freeze-dried and resuspended to 1.5 wt% in deionised water before being dispersed by sonication (Ultrasonic Processor, FB-505, Fisher – 550 W), at 30% amplitude with 1 s on 1 s off pulses, for ∼1 h or until the dispersion became transparent.

### Hydrophobic modification

OCNF fibrils were prepared as described above and dispersed at ∼0.5 wt% in water *via* probe sonication. Octylamine (Arcos Organic, +99%) was added in large excess (∼10×) and the pH corrected to 5 (with diluted hydrochloric acid solution, *ca.* 1 M). An equimolar amount of EDC (1-ethyl-3-(3-dimethylaminopropyl)carbodiimide hydrochloride) (Sigma, +99%)/NHS (*N*-hydroxysuccinimide) (Sigma, 98%) 1 : 1 solution was added dropwise and the pH of the mixture maintained at pH 5.

The reaction was left overnight and then 50 wt% propan-2-ol (BDH Chemicals, 100%) in water was added to the reaction mixture in a 2 to 1 ratio which causes the C8-OCNF to aggregate. The mixture was centrifuged at 5000 RCF (relative centrifugal force) for ten minutes and the precipitate (C8-OCNF) collected. The precipitate was washed twice more with 50 wt% propan-2-ol and centrifuged.

The precipitate was re-suspended in deionised water and dialysed against deionised water for 24 h to remove the propan-2-ol. The C8-OCNF was dialysed for a further 24 h against deionised water with a pH of 3 to remove any ionically bound amine. Further dialysis was performed against neutral deionised water for 72 h with regular replacement of the water to remove any remaining salts.

As with OCNF, the C8-OCNF was then freeze-dried and re-dispersed to the desired wt% using sonication.

The *ζ*-potential of C8-OCNF was measured using a Malvern Zeta-sizer Nano ZSP® (Malvern, UK) in a folded capillary electrode cell using ultrapure Milli-Q water as the dispersant. The sample was equilibrated at 25 °C for 120 s prior to testing and the results taken from an average of 3 measurements of 100 scans each.

### Starch

Starch dispersions were prepared by dissolving 1.5 wt% soluble starch (Sigma-Aldrich, S9765) in deionised water at 80 °C with stirring for 45 minutes, then allowing it to return to room temperature.

### Deep eutectic solvent

Menthol (Alfa Aesar, 99%) and dodecanoic acid (Acros, 98%) were combined in a 2 : 1 molar ratio and stirred at 50 °C until a homogenous liquid was formed.

### Emulsions

Aqueous dispersions were combined with the DES in a 70 : 30 volume ratio and then the samples were sonicated (Ultrasonic Processor, FB-505, Fisher – 550 W), at 20% amplitude with 1 s on 1 s off pulses, for 2 minutes. For polysaccharide-stabilised emulsions, the aqueous phase contained either starch (Sigma, Soluble S9765), OCNF or C8-OCNF at 1.5 wt%. For the surfactant-stabilised emulsions, the aqueous phase contained either 1 wt% Tween20 (8 mM) (Sigma) or 100 mM AOT (Acros, 96%) (4.5 wt%).

### Characterisation

Droplet measurements were made using a Mastersizer 3000E laser diffraction particle size analyser (Malvern, UK). Samples were added dropwise to the dispersion unit until the obscuration was within the acceptable range (7–20%). Five repeat measurements were made for each time point to ensure sample stability. Droplet size is reported as Sauter diameter *D*[3,2] for easy comparison.

SAXS measurements were performed on an Anton-Paar SAXSpoint 2.0 provided by the Material and Chemical Characterisation Facility (MC^2^)^33^ equipped with a copper source (Cu Kα, *λ* = 1.542 Å) and a 2D EIGER R series Hybrid Photon Counting (HPC) detector. The sample detector distance was 556.9 mm covering a *q* range of about 0.008–0.4 Å^−1^. The emulsions were loaded into 1 mm quartz capillaries and measured at 25 °C (±0.1 °C Peltier unit). Data was collected in one frame, with 900 s exposure, then processed. Fitting was performed using SASView (Version 4.2.1, see http://www.sasview.org/for more information). The model for elliptical cylinders was used from SASView 4.2.1 without modification.^34^ Fitting parameters are given in the ESI.‡

Viscosity of 1.5 wt% OCNF or C8-OCNF in water was measured using a stress-controlled Discovery Hybrid Rheometer, Model HR-3 (TA Instruments) with a sand-blasted 40 mm parallel plate geometry with a gap of ∼800 μm. Flow sweeps were performed at shear rates from 0.01 to 100 s^−1^ with ten points per decade, at 25 °C (Peltier unit, ±0.1 °C).

## Results and discussion

**Fig. 1:**
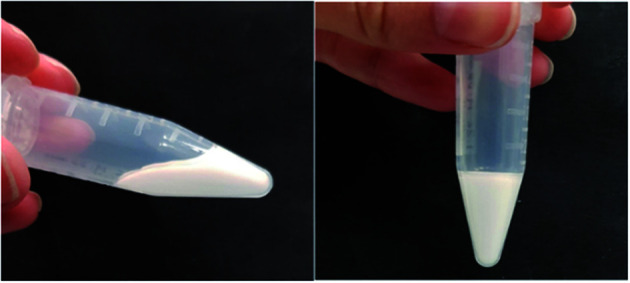
DES in water emulsion (30 : 70 volume ratio) stabilised by 1.5 wt% OCNF after more than 200 days at room temperature.

Emulsions made with OCNF remained visually stable for more than 200 days at room temperature with no evidence of aggregation or creaming (Fig. 1). Emulsions made with C8-OCNF creamed after 23 days, but the creamed layer remained stable (see Fig. S1‡). Creaming is due to the lower density of the DES droplets, compared to the bulk water.

**Fig. 2:**
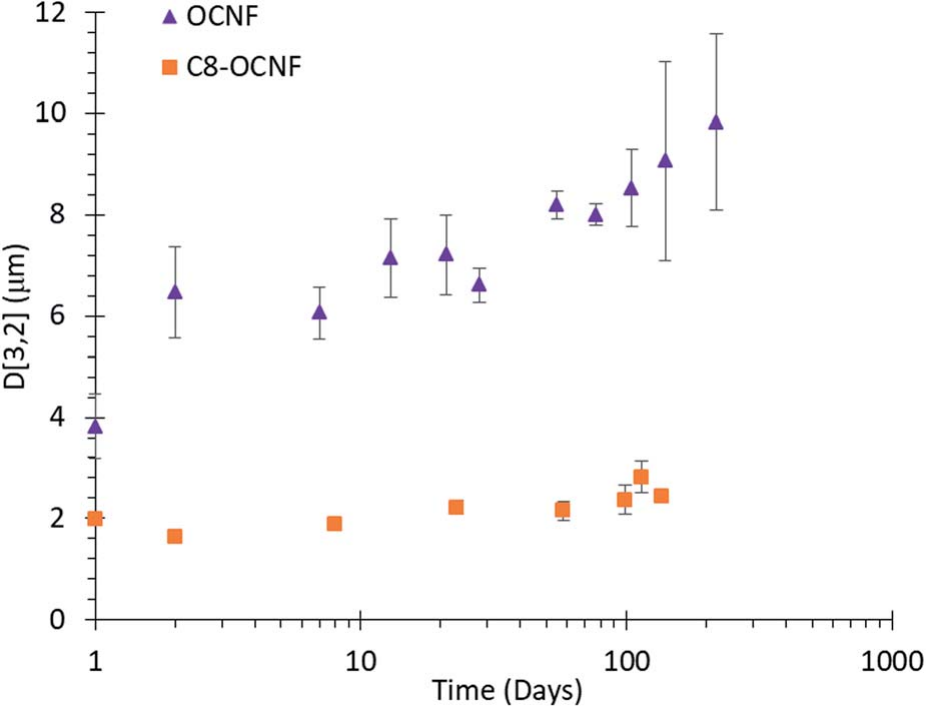
Average droplet size of DES in water (30 : 70 volume ratio) emulsions stabilised with either 1.5 wt% OCNF or C8-OCNF, error bars based on standard deviation of repeat measurements.

Visual observations were supported by laser diffraction measurements of droplet size. As shown in Fig. 2, droplets stabilised by OCNF remained stable in excess of 200 days with only a minor increase in droplet size. Droplets stabilized with C8-OCNF were smaller and appeared to be more stable, with almost no change even after 100 days.

The hydrophobic chains on C8-OCNF will allow greater interaction with the hydrophobic DES, compared to unmodified OCNF, and reduce the energy necessary to create a new interface, thus improving homogenization. This could allow tighter packing around the droplets, leading to smaller, more stable droplets. Similar effects have been observed for cellulose nanocrystal-stabilised emulsions using standard oils.^35,36^

While larger droplets may be expected to cream faster due to having a lower density, this was not the case here. The difference in creaming between OCNF and C8-OCNF is due to the higher viscosity of the continuous phase of OCNF- over C8-OCNF-stabilised emulsions which works to prevent creaming (Fig. S2‡).^22^ Increased viscosity comes from the higher surface charge of OCNF compared to C8-OCNF, which increases the excluded volume.

Previous emulsion research demonstrated that cellulose nanofibrils with higher charge provided greater stability than lower charged fibrils.^23^ The method of hydrophobisation used here necessitates the replacement of some of the negative carboxylate groups with hydrophobic chains, thus reducing the *ζ* potential from −60 mV to −43 mV. This would reduce repulsion between fibrils, and allow movement of droplets, ultimately leading to creaming.

In comparison, emulsions stabilised with soluble starch polymers, rather than cellulose particles, which have an even smaller *ζ* potential (−14 ± 1 mV)^37^ creamed on day 2. By day 23 there was precipitation and clumping and the mean droplet size was over 20 μm (Fig. S3‡).

**Fig. 3:**
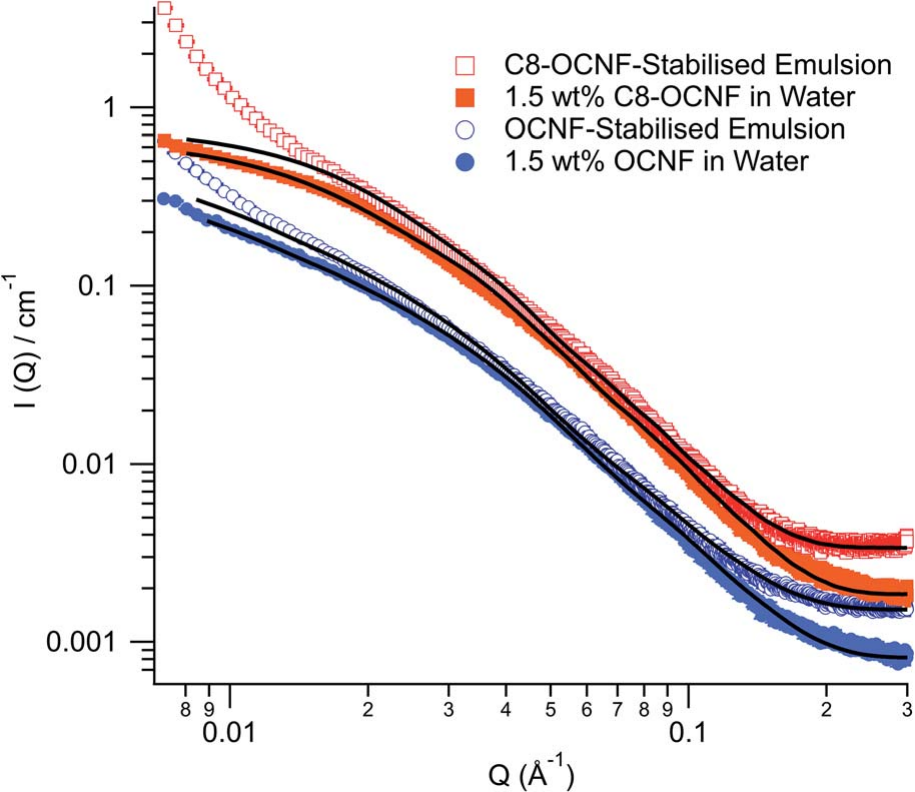
SAXS patterns of 1.5 wt% OCNF (filled circles) or C8-OCNF (filled squares) in water, and the DES in water emulsions stabilised by either 1.5 wt% OCNF (open circles) or C8-OCNF (open squares). Model fits are shown as black lines (parameters given in ESI‡).

Small angle X-ray scattering (SAXS) was used to investigate the structure of these emulsions. Fig. 3 shows the scattering patterns for OCNF and C8-OCNF in water and for the DES in water emulsions.

The scattering pattern of OCNF in water can be fit with an elliptical cylinder model with a minor radius of 12.4 ± 2 Å and a major radius of 58 ± 9 Å, in line with previous research.^32^ The same model can be used to fit the majority of the data for the emulsion sample, with a minor radius (13.4 ± 2 Å) and a major radius (66 ± 9 Å) that are within the error for OCNF fibrils in water, suggesting that the OCNF fibrils are dominating the SAXS signal.

The scattering pattern for C8-OCNF in water can be fit with the same parameters as OCNF except that the length is significantly shorter (300 ± 20 compared to >1000 Å). Given the benign nature of the modification procedure, this apparent change in length is most likely a result of fibril flocs. The length reported by SAXS is actually the distance between intersection points rather than an actual shortening of the fibrils (see Fig. S5‡). Similar aggregation has been observed for nanocrystalline cellulose upon addition of salt to screen repulsive charges.^38^ As with OCNF, the SAXS data from the emulsion stabilised by C8-OCNF can be fit with a similar model.

As shown in Fig. 3, the scattering from both emulsion samples have an upturn at low *q* which cannot be fitted with the elliptical cylinder model. This is probably from the emulsion droplets. Based on the laser diffraction data, these droplets are far outside of the probed range of this SAXS instrument and so cannot be fitted. The upturn appears to happen at higher *q* for the C8-OCNF stabilised emulsions, indicative of smaller droplets, which is consistent with the laser diffraction results. Also, due to the smaller surface charge, C8-OCNF is prone to self-aggregation, which could also contribute to the low-q signal.

The SAXS results demonstrate that for both OCNF and C8-OCNF the cellulose nanofibril structure is unchanged in the presence of the hydrophobic DES, compared to in water dispersions.

For the purposes of completeness, DES in water emulsions were also made with more traditional surfactants (dioctyl sodium sulfosuccinate (AOT) or Tween20). This produced emulsions with even smaller droplets (∼0.2–0.6 μm) that remained stable for more than 60 days (see ESI‡). However, the emulsion stabilised with AOT broke down by day 100, with separate oil and water layers. The emulsion stabilised with Tween20 creamed within two weeks but otherwise remained visually stable throughout the observation period (see ESI‡).

While the surfactant-stabilised emulsion appeared somewhat stable, the cellulose-stabilised pickering emulsions are of greater interest, not only due to prolonged stability (compared to AOT-stabilised) but also due to bioavailability and renewability of this stabiliser. Furthermore, particle-stabilised pickering emulsions are superior to surfactant-stabilised emulsions *e.g.* in terms of stability (*e.g.* of very large droplets, a wide range of oil/water ratios, and against Ostwald ripening) and the capacity for phase inversion.^39^

Particles can enhance emulsion stability not only by coating droplets like surfactants but also by forming a three dimensional network which immobilises droplets.^39–41^ The OCNF offers the added benefit of being a rheological modifier on its own.^42,43^ Thus, stabilization and rheological control can be achieved using the same additive.

Preliminary research by our group suggests that other polysaccharide nanoparticles (*e.g.* cellulose nanocrystals) are also capable of stabilising DES in water emulsions.

Future research will focus on using other hydrophobic DESs, or even making DES in DES emulsions, as well as exploring applications of DES-based emulsions.

## Conclusions

This research presents for the first time a DES in water emulsion stabilised by polysaccharides. DES in water emulsions offer new possibilities *e.g.* for chemical extraction and separation, controlled release (*e.g.* of drugs), as reactor systems, and for template-based synthesis *e.g.* of metal organic frameworks.

## Conflicts of interest

There are no conflicts to declare.

## Supplementary Material

RA-010-D0RA07575B-s001
