# Supplementary material for: Deep eutectic solvent in water pickering emulsions stabilised by cellulose nanofibrils
Source: RSC Adv. 2020 Oct 7;10(61):37023–7. doi: 10.1039/d0ra07575b (PMC9057056; doi:10.1039/d0ra07575b)
Supplement: RA-010-D0RA07575B-s001 [file RA-010-D0RA07575B-s001.pdf]

Electronic Supplementary Material (ESI) for RSC Advances.
This journal is © The Royal Society of Chemistry 2020

Deep Eutectic Solvent in Water Pickering Emulsions Stabilised by Cellulose Nanofibrils – Supplementary Information

Saffron J. Bryant,[a, b] Marcelo A. da Silva,[a] Kazi M. Zakir Hossain,[a] Vincenzo Calabrese,[a] Janet L. Scott,[a,c] Karen J. Edler[a*]*

[a] Department of Chemistry, University of Bath, Claverton Down, Bath, BA2 7AY, United Kingdom
[b] School of Science, RMIT University, Melbourne, Victoria, 3001, Australia
[c] Centre for Sustainable Chemical Technologies, University of Bath, Claverton Down, Bath, BA2 7AY, United Kingdom

saffron.bryant@rmit.edu.au, K.Edler@bath.ac.uk

Results and Discussion

Visual Observation

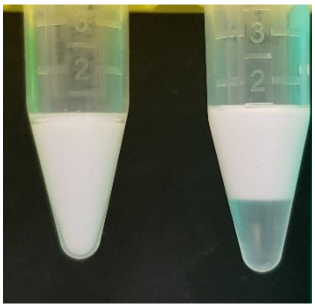

Figure S1. Emulsions stabilised with either OCNF (left) or C8-OCNF (right), demonstrating creaming of the C8-OCNF stabilised emulsion

Rheology

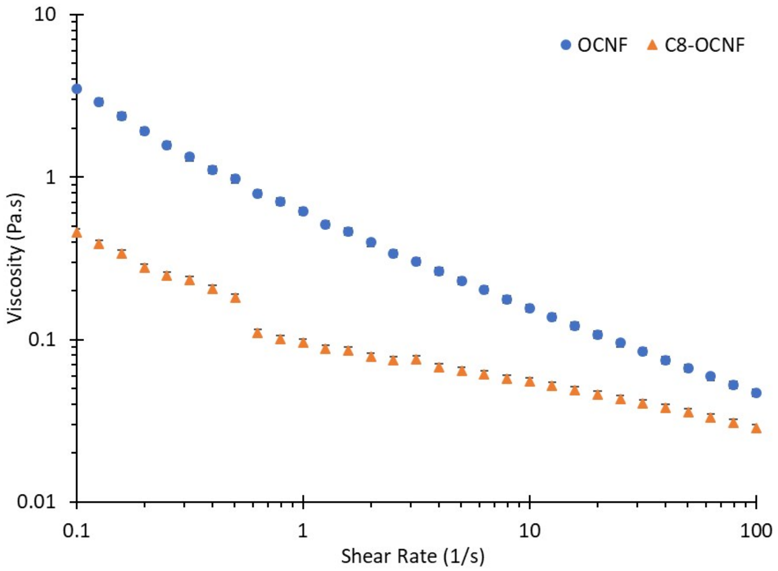

Figure S2. Flow sweep curves of 1.5 wt% OCNF or C8-OCNF in water

Droplet Size

As shown in Figure S3, the emulsions stabilised by starch had a much greater increase in droplet size compared to those stabilised by OCNF or C8-OCNF (the data for OCNF and C8-OCNF are duplicated here from the main manuscript for comparison). This coincided with visible instability and syneresis of the starch emulsion which did not occur in the other two.

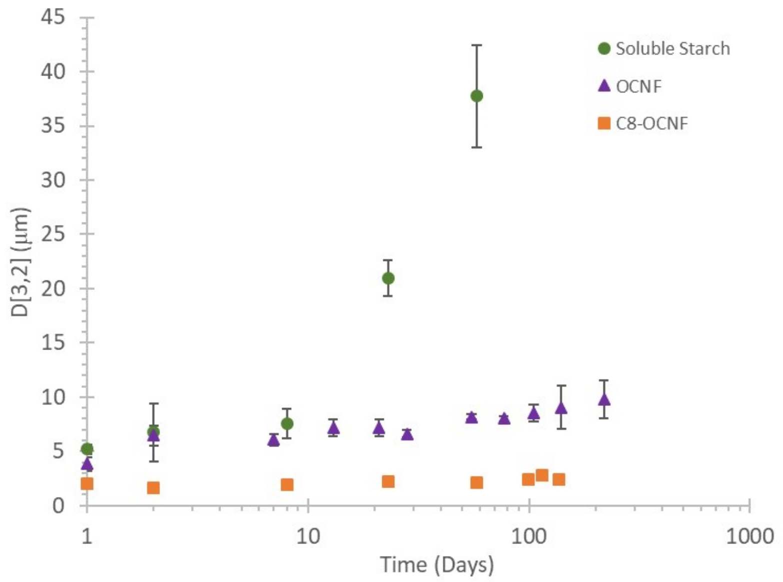

Figure S3. Droplet size of menthol:dodecanoic acid in water emulsions stabilised by either starch, OCNF, or C8-OCNF.

Emulsions stabilised with surfactant (either AOT or Tween20) had much smaller droplet sizes than those stabilised with polysaccharides.

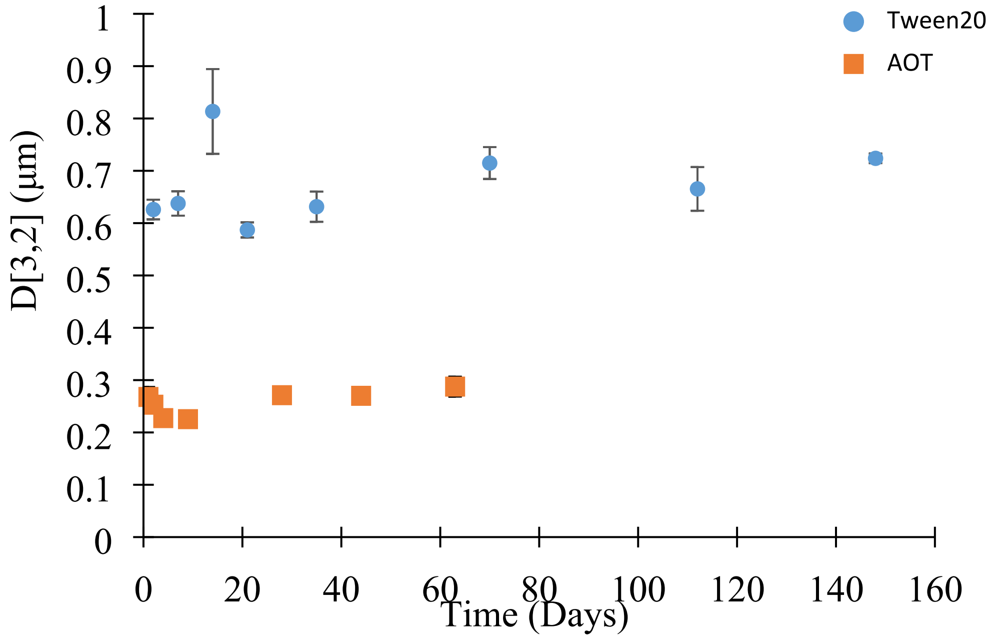

Figure S4. Droplet size of a menthol:dodecanoic acid in water emulsions stabilised with either Tween20 or AOT

SAXS

Fitting parameters for the fits shown in Figure 3 are given in Table S1. In all cases an elliptical cylinder model was used.

Table S1. Fitting parameters for OCNF and C8-OCNF in water and as part of an emulsion

**Sample**	**Minor Radius (Å)**	**Major Radius (Å)**	**Length (Å)**
1.5 wt% OCNF in H_2O	12.5±2	59±9	>1000
DES in Water, OCNF stabilised	13.4±2	66±10	>1000
1.5 wt% C8-OCNF in H_2O	13.5±2	64±9	305±20
DES in Water, C8-OCNF stabilised	15.5±2	70±9	266±20

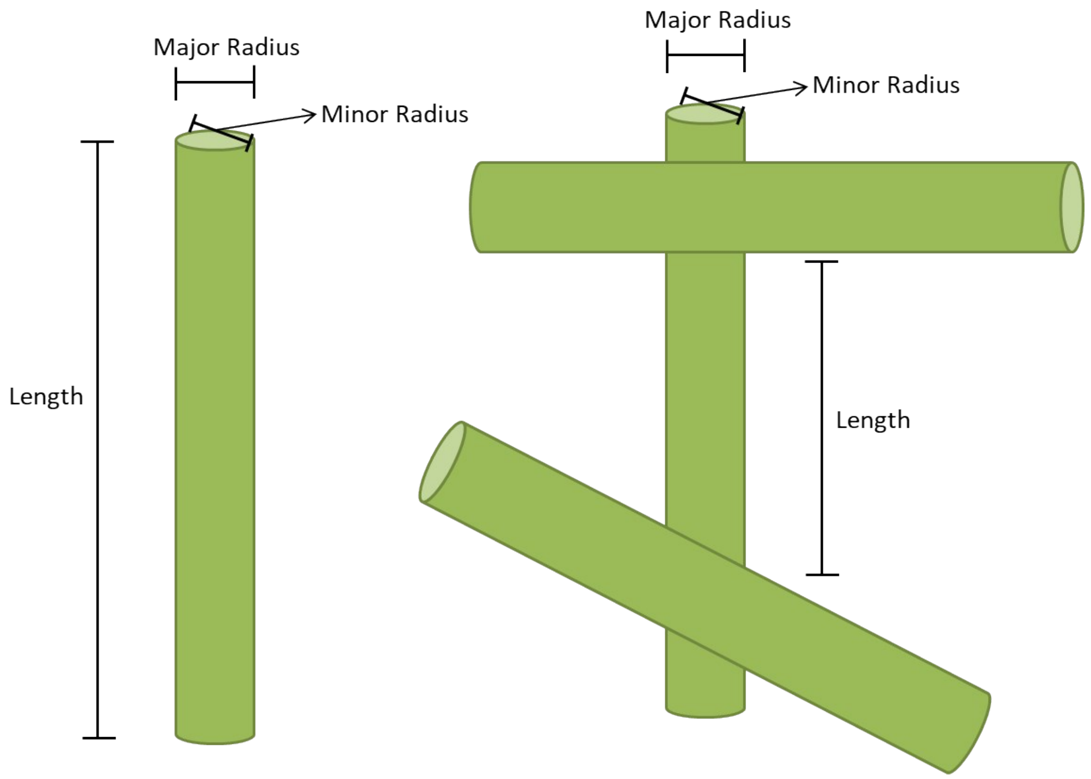

Figure S5. Explanation for apparent shortened length of C8-OCNF (right) compared to OCNF (left) as a result of fibril floculation.
